# Ginsenoside Rb1 Ameliorates Age‐Related Cognitive Impairment Potentially by Modulating the NF‐κB Signaling Pathway

**DOI:** 10.1002/brb3.71334

**Published:** 2026-03-30

**Authors:** Wei‐Feng Lu, Yu‐Zhen Zhang, Zhi‐Gang Chen, Qun‐Lin Xiao, Shi‐Ye Ke

**Affiliations:** ^1^ Department of Cardiology, the Eighth Affiliated Hospital Sun Yat‐sen University Shenzhen Guangdong Province People's Republic of China; ^2^ Department of Neurology, the Fifth Affiliated Hospital Sun Yat‐sen University Zhuhai Guangdong Province People's Republic of China

**Keywords:** aged mice, cognitive impairment, ginsenoside Rb1, NF‐κB signaling pathway

## Abstract

**Introduction:**

Age‐related cognitive impairment affects the quality of life of the elderly, contributing to a substantial healthcare burden. Ginsenoside Rb1 (Rb1), an active component of ginseng, has been shown to possess various biological functions, including antisenescence, anti‐inflammatory, and neuroprotection effects. This study investigated whether Rb1 attenuates age‐related cognitive impairment and aimed to elucidate the relevant molecular mechanism.

**Methods:**

Female C57BL/6J mice (aged 2 and 18 months) received daily intraperitioneal injections of either Rb1 or vehicle for 3 months. Subsequently, their cognitive ability was assessed using the Morris water maze (MWM) test. Pathological changes in hippocampal neurons were investigated using hematoxylin and eosin (HE) staining and Nissl staining. In addition, Western blot and immunohistochemistry were employed to examine proteins relevant to cellular senescence (cyclin‐dependent kinase inhibitor 1A [p21^Cip1^] and cyclin‐dependent kinase inhibitor 2A‌ [p16^INK4a^]), inflammation (interleukin‐6 [IL‐6] and tumor necrosis factor‐α [TNF‐α]), and markers indicating nuclear factor‐kappa B (NF‐κB) pathway activation in hippocampus tissue.

**Results:**

In the MWM test, Rb1 treatment significantly ameliorated cognitive dysfunction in aging mice compared to controls. Results from HE staining and Nissl staining showed marked neuronal loss, neurodegeneration, and uneven cytoplasm distribution of Nissl bodies in the hippocampus of aging mice, all of which were alleviated by Rb1 treatment. Immunohistochemistry and Western blot analyses demonstrated a pronounced increase in p21^Cip1^, ‌p16^INK4a^, IL‐6, and TNF‐α expression levels in the CA1 and CA3 regions of the hippocampus, along with enhanced phosphorylation of NF‐κB, inhibitor of NF‐κB (IκB) kinase (IKKβ) and IκBα in the aging mice. However, Rb1 treatment significantly downregulated p21^Cip1^, p16^INK4a^, IL‐6, and TNF‐α expression levels, indicating that Rb1 alleviated cellular senescence and neuroinflammation in the hippocampus. Furthermore, Rb1 administration evidently repressed the phosphorylation of NF‐κB, IKKβ, and IκBα in the aging mice.

**Conclusion:**

Rb1 may alleviate aging‐related cognitive impairment by suppressing hippocampal inflammation, potentially through modulation of the NF‐κB signaling pathway.

## Graphical abstract

Ginsenoside Rb1 may alleviate age‐related cognitive impairment by suppressing hippocampal inflammation, potentially through modulation of the NF‐κB signaling pathway.

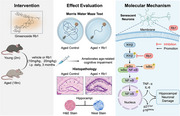

## 1 Introduction

Advanced age alone is a major risk factor for chronic diseases, including neurodegenerative disorders characterized by ongoing neuronal loss and dysfunction, contributing to a growing public health burden (Hou et al. 2019; Kwon et al. 2020). The aging process frequently involves the progressive deterioration of learning abilities, attention, and memory formation (Bishop et al. 2010). These cognitive deficits manifest with varying severity, from occasional memory lapses to extensive impairment across multiple domains. Memory problems are often the earliest indicators of age‐related cognitive decline and typically intensify as the condition advances (Dahan et al. 2020). There are few effective existing treatments for aging‐related neurodegenerative diseases, which generally progress irreversibly and impose a substantial healthcare burden in aging societies (Kwon et al. 2020). Consequently, it is crucial to elucidate the underlying mechanisms and identify potential therapeutic agents for reducing the incidence and prevalence of aging‐related neurodegenerative diseases.

Episodic memory involves learning and recalling associations between items and their spatiotemporal context. These intricate processes occur primarily in the hippocampus, a small, seahorse‐shaped structure within the medial temporal lobe, which comprises subfields known as the cornu ammonis (CA) regions. The structural and functional integrity of the hippocampus, which is crucial for learning and memory consolidation, becomes impaired with advancing age (Dahan et al. 2020). The aging brain is characterized by several hallmarks associated with increased susceptibility to neurodegenerative diseases. These markers include genomic instability, telomere attrition, epigenetic alterations, loss of proteostasis, mitochondrial dysfunction, cellular senescence, deregulated nutrient sensing, stem cell exhaustion, and altered intercellular communication (Hou et al. 2019). However, the precise mechanisms underlying hippocampal functional loss in episodic memory remain unclear. Compelling experimental evidence has implicated age‐related chronic neuroinflammation as a critical factor in the initiation and progression of hippocampal impairment, ultimately contributing to the pathogenesis of cognitive dysfunction (Geinisman et al. 1995; Martínez de Toda et al. 2021). Upon activation, glial cells secrete toxic inflammatory mediators, including cytokines and chemokines (e.g., tumor necrosis factor‐α [TNF‐α] and interleukin‐6 [IL‐6]), which drive neuroinflammation and are directly or indirectly implicated in the development of neurodegenerative diseases (Kwon and Koh 2020).

Nuclear factor κB (NF‐κB) is a transcription factor that mediates complex biological processes, including cell survival and inflammation (Ridder and Schwaninger 2009; Zheng et al. 2025). Under normal conditions, NF‐κB remains inactive in the cytoplasm by binding to its regulatory protein inhibitor (IκB). When the NF‐κB signaling pathway is activated, IκB kinase (IKK) phosphorylates IκB, targeting it for degradation and releasing NF‐κB for nuclear translocation, where it regulates the expression of multiple target genes (Dolatshahi et al. 2021; Lawrence 2009). Selective NF‐kB inhibition can suppress subsequent proinflammatory gene expression, thereby reducing the overall inflammatory response in the brain (Lawrence 2009). Prior research using aged animal models demonstrates that hippocampal neuroinflammation and NF‐kB signaling may contribute to cognitive postoperative dysfunction (Li et al. 2023; Qian et al. 2023). A recent study further revealed that NF‐kB inhibition attenuates neuronal damage and promotes apoptosis of senescent neurons, thereby facilitating the clearance of damaged cells and ultimately alleviating age‐related cognitive decline (Liang et al. 2025). These findings highlight the important role of the NF‐kB signaling pathway in age‐related neurodegenerative disorders.

Accordingly, agents that inhibit inflammation may attenuate age‐associated cognitive impairment. Numerous natural medicines with strong anti‐inflammatory properties have been identified as beneficial for the treatment of neurodegenerative diseases (Kempster and Ma 2022; Rauf and Rahman 2022). Accumulating data suggest that ginsenoside Rb1 (Rb1), one of the most active components of ginseng, possesses multiple biological functions, including antisenescence, anti‐inflammatory, and neuroprotective effects (Zhou et al. 2019). The literature reports that Rb1 benefits the central nervous system by improving learning and memory and by preventing and treating dementia (Lin et al. 2019; Wang et al. 2018). Recent studies have further revealed that Rb1 can rescue cisplatin‐induced memory impairment by restoring neuronal activity through reducing oxidative stress and neuroinflammation, and by recovering cholinergic neuron function (Chen et al. 2019). Another study demonstrated that Rb1 treatment can prevent scopolamine‐induced amnesia and enhance memory by inhibiting neuroinflammation (Yang et al. 2020). This research on Rb1's neuroprotective effects has focused on broader anti‐inflammatory or antioxidant mechanisms. Our previous work showed that Rb1 alleviates aging‐related myocardial dysfunction by suppressing inflammation via regulation of the NF‐κB signaling pathway (Ke et al. 2020). However, no published research has examined the protective effects and mechanisms by which Rb1 counteracts age‐associated cognitive impairment by inhibiting neuroinflammation during the natural aging process. In addition, no published study has linked Rb1's cognitive benefits for natural aging to the modulation of hippocampal NF‐κB signaling, a key regulator of neuroinflammation.

We hypothesize that Rb1 treatment alleviates age‐related cognitive deficits by inhibiting neuroinflammation, potentially through regulation of the NF‐κB signaling pathway. We used a mouse model of aging‐induced cognitive dysfunction to investigate the effects of Rb1 on cognitive impairment and its underlying mechanisms in the hippocampus. Unlike previous studies, which have primarily focused on disease models or artificial interventions, our work directly targets the natural aging process to more accurately reflect the roles of the NF‐κB signaling pathway and the Rb1 agent under physiological conditions. This provides a new perspective on the molecular mechanisms underlying aging‐related cognitive impairment, offering a potential strategy to delay age‐associated cognitive decline.

## 2 Materials and Methods

### 2.1 Experimental Reagents

**FIGURE 1:**
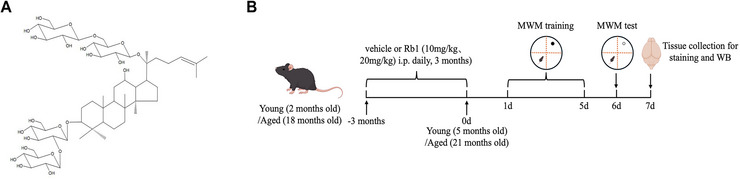
**Experimental design schematic**. (A) The chemical structure of Rb1. (B) Experimental design schematic. Young (2 months) and aged (18 months) mice received daily intraperitoneal injections of either vehicle or Rb1 (10 or 20 mg/kg) for three months, followed by euthanasia at 5 months of age and 21 months of age, respectively. From Day 1 to Day 6, spatial memory was assessed using the MWM training and testing protocol. On Day 7, all mice were euthanized, and hippocampal tissues were collected for subsequent histological and molecular analyses.

Rb1 (molecular weight [MW] = 1109 kD; molecular structure shown in Figure 1A; purity greater than 98%) was obtained from Victory (Sichuan, China). Antibodies against TNF‐α (11948S) and GAPDH (5174S), and an NF‐κB pathway sampler kit (9936) were purchased from Cell Signaling Technology (Beverly, MA, USA). Antibodies against cyclin‐dependent kinase inhibitor 1A (p21^Cip1^) (ab188224) and ‌cyclin‐dependent kinase inhibitor 2A‌ (p16^INK4a^) (ab51243) were purchased from Abcam (Cambridge, MA, USA). Antibodies against IL‐6 (21865) were purchased from Proteintech Group (Chicago, MA, USA). All other reagents used in the study were of analytical grade.

### 2.2 Animals and Management

This study was approved by the Institutional Animal Care and Use Committee (IACUC) of Sun Yat‐Sen University. Female animals exhibit increased susceptibility to age‐related cognitive decline, with an earlier onset and more rapid progression of neurodegenerative disease (Gao et al. 2016; Hebda‐Bauer et al. 2007). Furthermore, longevity studies predominantly use female C57BL/6 mice because they are less prone to aggression during long‐term group housing, thereby minimizing stress‐ and injury‐related confounds (Hsu et al. 2018; Qin et al. 2018). For these reasons, we used female C57BL/6 mice in our long‐term investigation. We obtained young (2 months old) and aged (18 months old) female C57BL/6J mice from the Center of Experimental Animals, Sun Yat‐Sen University. Mice were housed, five per cage, under controlled conditions (24 ± 2°C and 50 ± 5% humidity) under a 12–12 h light–dark cycle under specific pathogen‐free conditions, and were fed a normal diet. Twenty young female C57BL/6J mice (Young group) received a daily intraperitoneal injection of vehicle (sterile saline solution) for 3 months before undergoing euthanasia at 5 months of age. The Rb1 dose was selected according to established protocols from the literature and prior experiments in our laboratory, which have demonstrated that these doses are effective and safe in murine neurological and cardiovascular studies (Ke et al. 2020; Yu et al. 2024). Sixty aged female C57BL/6J mice were randomly assigned to Old + vehicle, Old + 10 mg/kg Rb1 (Old + Rb1‐10) and Old + 20 mg/kg Rb1 (Old + Rb1‐20) groups (*n* = 20 each). The mice that survived until the end of the experiment received daily intraperitoneal injections of either vehicle or Rb1 (10 or 20 mg/kg) for 3 months, after which they were euthanized at 21 months of age. During the treatment period, a total of 5 mice were lost across all groups due to severe weight loss or non‐treatment causes. These included 2 mice in the Old + vehicle group, 2 in the Old + Rb1‐10 group, and 1 in the Old + Rb1‐20 group. No animals were excluded based on post‐treatment behavioral or biochemical assessments. The schematic diagram of the experimental design is presented in Figure 1B.

### 2.3 Morris Water Maze Test

The Morris water maze (MWM) test is a classical and reliable behavioral task used to assess spatial learning and memory in rodents (Haider and Tabassum 2018). Young and aged mice in each group underwent the MWM to assess cognitive performance (Huang et al. 2025). The experimental equipment comprised a circular tank pool 150 cm in diameter and 50 cm in height. During the experiment, the pool was filled with opaque water containing a white, nontoxic dye at a depth of 30 cm and maintained at 25 ± 1°C. The pool was separated into four quadrants, with a 10 cm diameter escape platform placed 1 cm below the water's surface and in the center of one of the quadrants. The visual cues surrounding the pool were arranged to provide spatial orientation within the testing room and remained unchanged for the duration of the experiment. All mice underwent hidden‐platform training for 5 days (4 trials per day, 90 s per trial). For each trial, the mice were released from four starting quadrant positions in a different order and allowed to swim for 90 s. If a mouse spent less than 90 s exploring before finding the platform, the trial was terminated, and the time to find the hidden platform was treated as the escape latency. If the mouse failed to find the platform within the allotted time, it was guided to the platform and allowed to remain there for 15 s, and an escape latency of 90 s was recorded. The escape latencies for each mouse across the four trials were averaged prior to statistical analysis. On the sixth day of the exploration test, the platform was removed from the pool. Each mouse was permitted to swim freely for 90 s, starting from the diagonal quadrant opposite the original platform location. The total distance swum and the number of times each mouse crossed the former platform site were recorded. All experimental procedures were performed blind to groups.

### 2.4 Tissue Extraction

All test mice were anesthetized with a diethyl ether mask and were sacrificed immediately after the final MWM test. The whole brains of 5 mice were carefully removed and placed in 4% brain buffer. These samples were embedded in paraffin and coronally sectioned at 5 µm thickness. The sections were mounted on slides for subsequent hematoxylin‐eosin (HE) and Nissl staining. Whole brains from an additional 5 mice were also removed, placed in 4% brain buffer, paraffin‐embedded, and coronally sectioned at 5 µm. The resulting sections were mounted on slides for immunohistochemistry. The brains of the remaining 8–10 mice in each group were promptly removed on ice, and the hippocampi were rapidly dissected, frozen in liquid nitrogen, and stored at −80°C for Western blot analysis.

### 2.5 HE Staining

Hippocampus sections embedded in paraffin were stained with hematoxylin for 2 min. After washing and treatment with 1% acidic alcohol, the sections were counterstained with eosin for 3 min, then washed, dehydrated, and cleared in xylene prior to microscopic examination. Images of hippocampal sections were acquired by a blinded investigator using an optical microscope.

### 2.6 Nissl Staining

The paraffin‐embedded hippocampus sections were deparaffinized and rehydrated, followed by three 5‐min rinses in distilled water. These sections were stained for 5 min at 37°C with a Nissl staining solution (Beyotime, C0117, Shanghai, China). Subsequently, the samples were washed for 5 min each in 95% ethyl alcohol and xylene. Having been sealed with neutral balsam, the slides were examined under an optical microscope. Random HE‐ and Nissl‐stained sections were imaged, and the number of viable neurons was then counted and analyzed by technicians blinded to the experimental conditions.

### 2.7 Immunohistochemistry

Following deparaffinization and rehydration, the hippocampal tissue sections were subjected to antigen retrieval using microwave heating in citrate buffer (pH 6.0) for two 10‐min cycles. Nonspecific binding sites were blocked using 10% goat serum (Gibco, BRL, NY, USA) for 1 h at 37°C, after which the sections were incubated overnight at 4°C in a humidified chamber with primary antibodies targeting p21^Cip1^, p16^INK4a^, IL‐6, and TNF‐α. Following three phosphate buffer saline washes, the sections were incubated with horseradish peroxidase (HRP)‐conjugated secondary antibodies (Abcam) for 1 h at 37°C and then stained with diaminobenzidine (1:100, Abcam). Cell nuclei were counterstained with hematoxylin. Finally, the sections were mounted with neutral gum and imaged by a blinded investigator using a Zeiss microscope.

### 2.8 Western Blotting

Hippocampal tissues were homogenized in RIPA lysis buffer (HaiGene, Haerbin, China) containing a protease inhibitor cocktail (MedChemExpress, Monmouth Junction, NJ, USA) using ultrasonic disruption on ice. The lysates were centrifuged at 12,000 × *g* for 15 min at 4°C, and the resulting supernatant was collected. We quantified protein concentrations in the supernatant using a BCA assay kit (Beyotime Institute of Biotechnology, Jiangsu, China). Equal amounts of protein from each sample were separated using 6%–10% SDS‐PAGE, before being transferred to PVDF membranes (EMD Millipore, Billerica, MA, USA). After blocking with 5% bovine serum albumin (Gibco) for 1 h at room temperature, the membranes were incubated with primary antibodies against p21Cip1 (1:1000), p16INK4a (1:1000), TNF‐α (1:1000), IL‐6 (1:1000), p‐NF‐κB (1:1000), p‐IKKβ (1:1000), p‐IκBα (1:1000), NF‐κB (1:1000), IKKβ (1:1000), IκBα (1:1000), or GAPDH (1:1000). Proteins were detected using HRP‐conjugated IgG (Boster, Wuhan, China) and visualized using an ECL kit (EMD Millipore). Band intensities were quantified using ImageJ software (version 1.41; National Institutes of Health, MA, USA) to determine the relative expression levels of the target proteins. GAPDH served as the internal loading control.

### 2.9 Statistical Analysis

All experiments were performed in at least three independent replicates. Data are presented as mean ± SD. Statistical analyses were conducted using GraphPad Prism (version 10.1; San Diego, CA, USA). The MWM training phrase data were analyzed by a two‐way analysis of variance (ANOVA). For other data, comparisons among multiple groups were conducted using one‐way ANOVA. Post hoc comparisons among individual groups were performed using Tukey's test. Statistical significance was defined as *p* < 0.05.

## 3 Results

### 3.1 Rb1 Improved Cognitive Deficits in Aging Mice

**FIGURE 2:**
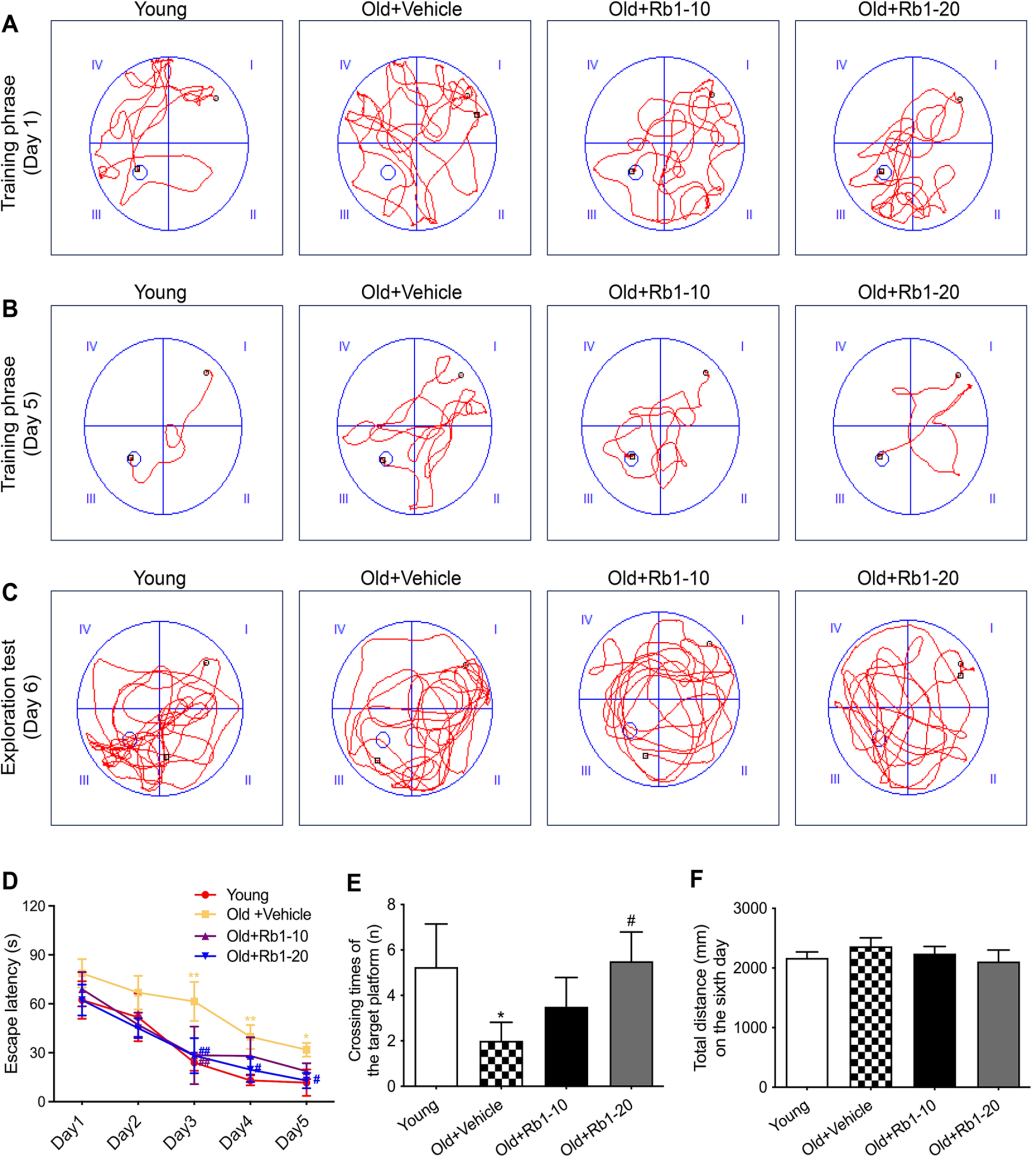
**Effects of Rb1 on aging‐related cognitive impairment assessed using the MWM test**. Representative search paths of mice on training phase Day 1 (A), Day 5 (B), and exploration test Day 6 (C). (D) Escape latency for locating the platform across training stages is shown. (E) The number of platform crossings on Day 6 is illustrated. (F) Motor ability was assessed by measuring total movement distance on Day 6. *n* = 10 mice per group. Data are expressed as mean ± SD (^*^ *p* < 0.05, ^**^ *p* < 0.01 vs. the Young group; ^#^ *p* < 0.05, ^##^ *p* < 0.01 vs. the Old + Vehicle group). Statistical analysis was performed using wo‐way ANOVA (D) and one‐way ANOVA (E, F), followed by Tukey's multiple comparisons tests.

The MWM test assessed the effect of Rb1 on spatial learning and memory deficits in aging mice. During the five‐day training phase, aged mice exhibited impaired spatial learning relative to young mice; however, Rb1 treatment reduced escape latency in the aged group (Figure 2B,D). The platform was removed on the sixth day to evaluate spatial memory. Aged mice crossed the former platform location less frequently than young mice, but Rb1 treatment, particularly at 20 mg/kg, alleviated this cognitive impairment by increasing the number of crossings (Figure 2C,E). To exclude the possible influence of a motor deficit in the MWM test, the total distance was examined. There were no notable differences in the distance traveled by the mice among the groups (Figure 2F). These results indicate that Rb1 ameliorates age‐related cognitive deficits in aging mice.

### 3.2 Rb1 Alleviated Senescence in the Hippocampi of Aging Mice

**FIGURE 3:**
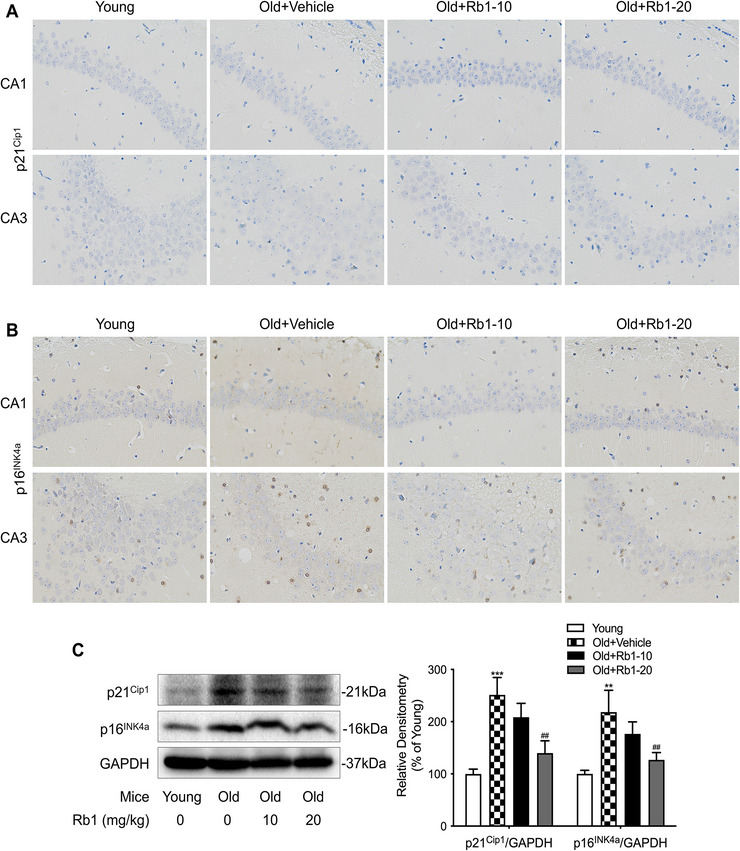
**Effect of Rb1 on hippocampal senescence**. Immunohistochemical staining images (magnification 400×) show p21^Cip1^ (A) and p16^INK4a^ (B) expression in mouse hippocampal CA1 and CA3 regions, *n* = 5 mice per group. (C) Western blot analysis of hippocampal p21^Cip1^ and p16^INK4a^ expression, *n* = 4 mice per group. Data are expressed as mean ± SD (^**^ *p* < 0.01, ^***^ *p* < 0.001 vs. the Young group; ^##^ *p* < 0.01 vs. the Old + Vehicle group). Statistical analysis was performed using one‐way ANOVA, followed by Tukey's multiple comparisons test.

To investigate the anti‐aging effects of Rb1, we assessed cellular senescence, a state characterized by the expression of proteins involved in cell‐cycle inhibition and irreversible growth arrest (Yang et al. 2019). The immunohistochemistry results (shown in Figure 3A,B) demonstrated that aging accelerated upregulation of p21^Cip1^ and p16^INK4a^ in the CA1 and CA3 hippocampal regions of mice in the Old+Vehicle group compared with the Young group. Rb1 treatment alleviated this hippocampal cellular senescence, with the most pronounced effect observed in the Old+Rb1‐20 group. Western blot analysis corroborated the immunohistochemistry findings, showing a more than 2‐fold increase in p21^Cip1^ and p16^INK4a^ protein expression in hippocampal tissues from aged mice relative to young mice. Following treatment with 20 mg/kg Rb1, the protein levels of p21^Cip1^ and p16^INK4a^ rose by only 39.90% and 26.80% compared to those in the Young group, which was significantly lower than the levels observed in the Old + Vehicle group (Figure 3C).

### 3.3 Rb1 Inhibited Hippocampal Neuronal Damage in Aging Mice

**FIGURE 4:**
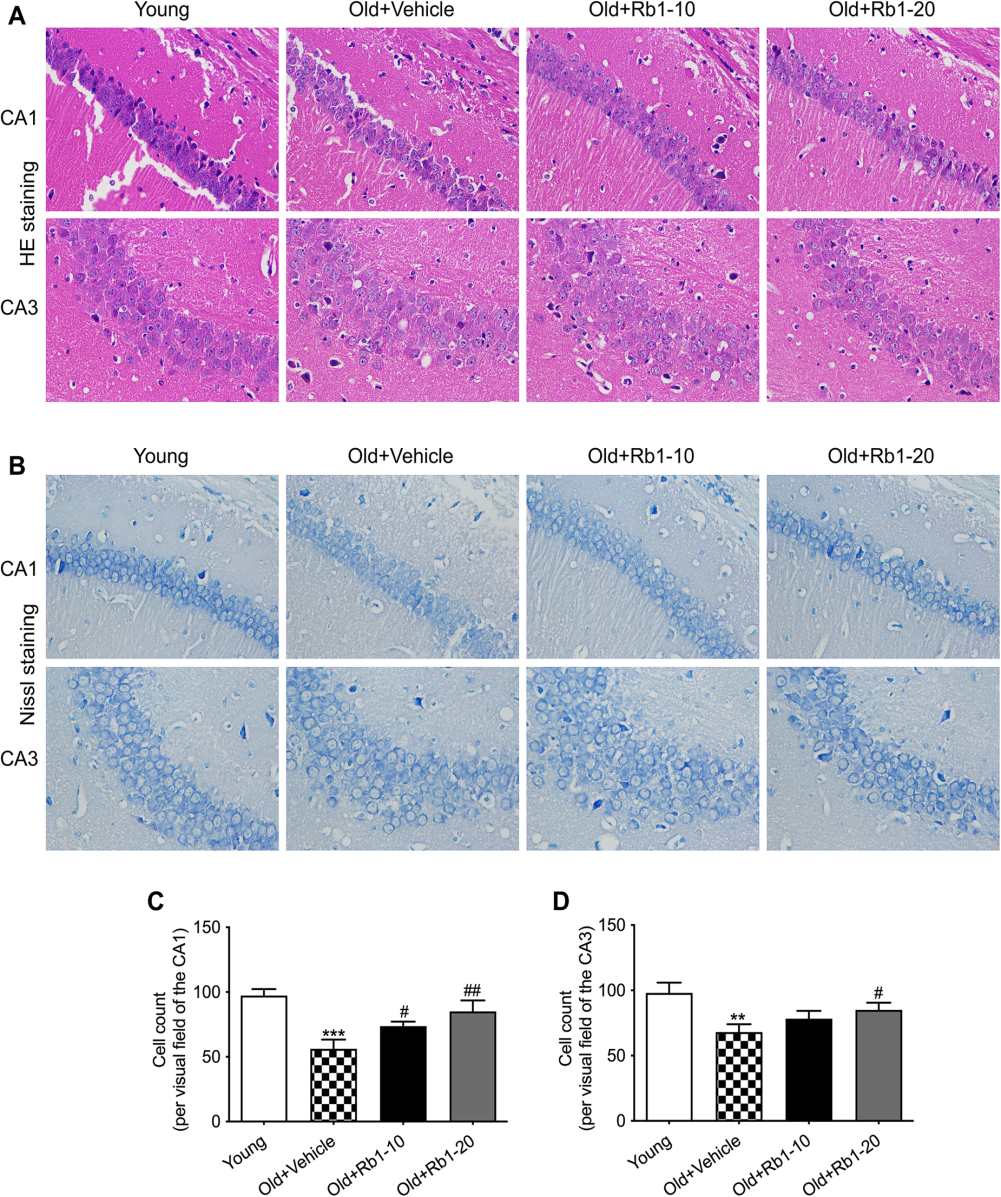
**Rb1 attenuated age‐associated hippocampal neuronal damage**. (A) Representative HE staining images from the hippocampal CA1 and CA3 regions (magnification 400×). (B) Representative Nissl staining images from the hippocampal CA1 and CA3 regions (magnification 400×). The number of living neurons in the hippocampal CA1 regions (C) and CA3 regions (D). *n* = 5 mice per group. Data are expressed as mean ± SD (^**^ *p* < 0.01 vs. the Young group; ^#^ *p* < 0.05, ^##^ *p* < 0.01 vs. the Old + Vehicle group). Statistical analysis was performed using one‐way ANOVA, followed by Tukey's multiple comparisons test.

We further assessed hippocampal tissue structure in mice using HE staining and Nissl staining. Representative images from the hippocampal CA1 and CA3 regions are presented in Figure 4A,B. Neuronal survival counts for these regions are shown in Figure 4C,D. Hippocampal neurons in young mice were densely packed with large nuclei and distinct nucleoli, and Nissl bodies were clearly visible and uniformly distributed throughout the cytoplasm. In contrast, aging mice exhibited marked neuronal loss and neurodegeneration, characterized by loosely organized neurons, uneven cytoplasmic staining, and significantly reduced neuronal survival within the CA1 and CA3 regions. Rb1 treatment alleviated these morphological alterations, particularly in the Old + Rb1‐20 group, indicating that Rb1 reduces neuronal cell loss.

### 3.4 Rb1 Attenuated the Aging‐Induced Inflammatory Response in Hippocampus

**FIGURE 5:**
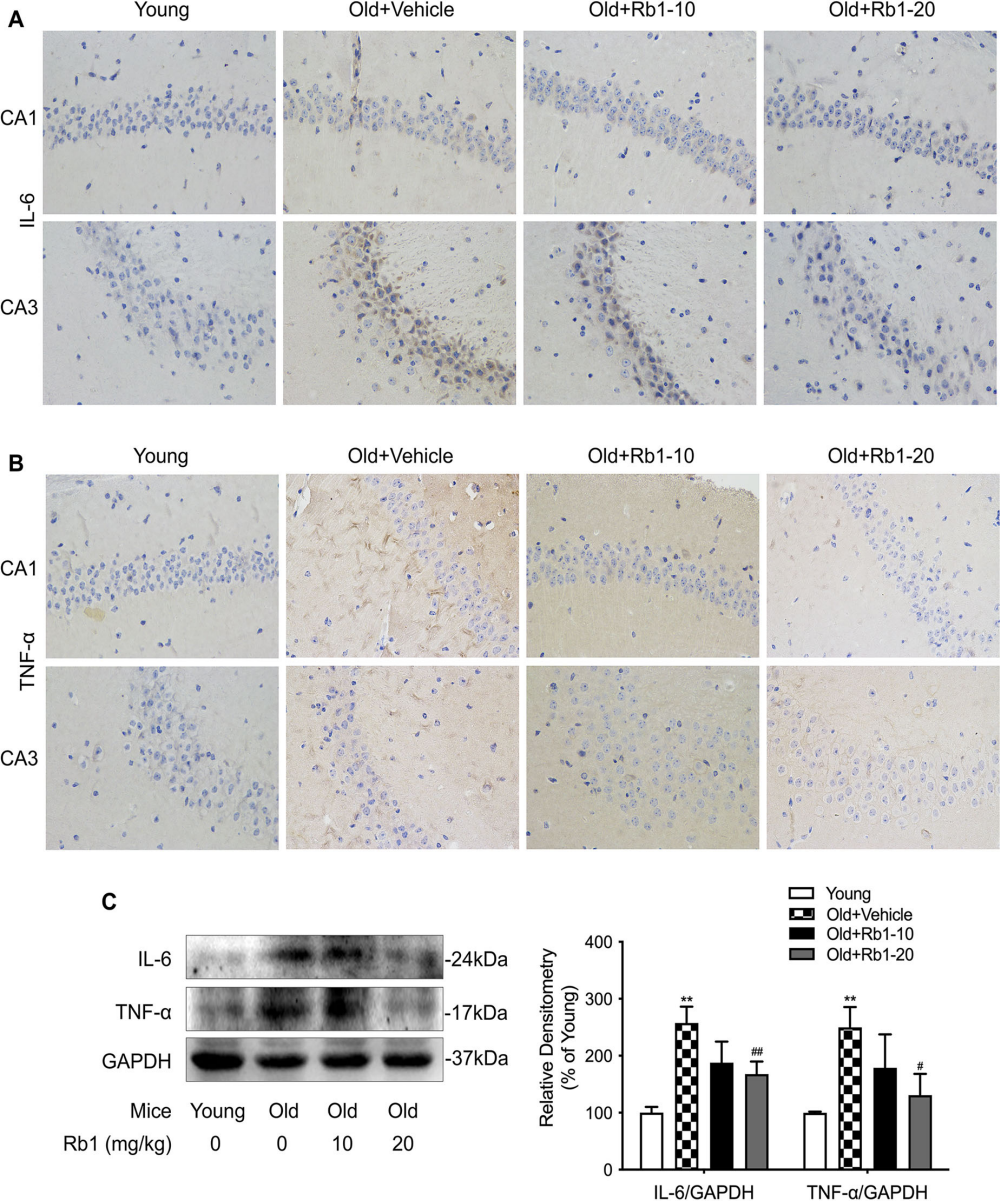
**Protective effects of Rb1 against proinflammatory reactions**. Immunohistochemical staining images (magnification 400×) depict IL‐6 (**A**) and TNF‐α (B) in the hippocampal CA1 and CA3 region of mice, *n* = 5 mice per group. (C) Western blot analysis of IL‐6 and TNF‐α expression in hippocampus tissue, *n* = 4 mice per group. Data are expressed as mean ± SD (^**^ *p* < 0.01 vs. the Young group; ^#^ *p* < 0.05, ^##^ *p* < 0.01 vs. the Old + Vehicle group). Statistical analysis was performed using one‐way ANOVA, followed by Tukey's multiple comparisons test.

Chronic inflammation is increasingly recognized as a contributor to aging‐related cognitive deficits (Ridder and Schwaninger 2009). To evaluate the anti‐inflammatory effects of Rb1 in aging mice, we measured relevant inflammatory mediators. Immunohistochemistry revealed that aging increased IL‐6 and TNF‐α expression in the hippocampal CA1 and CA3 regions of the Old + Vehicle group compared with the Young group (Figure 5A,B). Rb1 treatment mitigated this neuroinflammation, with the most pronounced effect observed in the Old+Rb1‐20 group. Consistent with these findings, Western blot analysis revealed a significant increase in hippocampal IL‐6 and TNF‐α protein levels in aged mice relative to those in young mice. The administration of 20 mg/kg Rb1 significantly reduced these protein levels compared to the Old + Vehicle group (Figure 5C).

### 3.5 Rb1 Suppressed NF‐κB Signaling Pathways Activation

**FIGURE 6:**
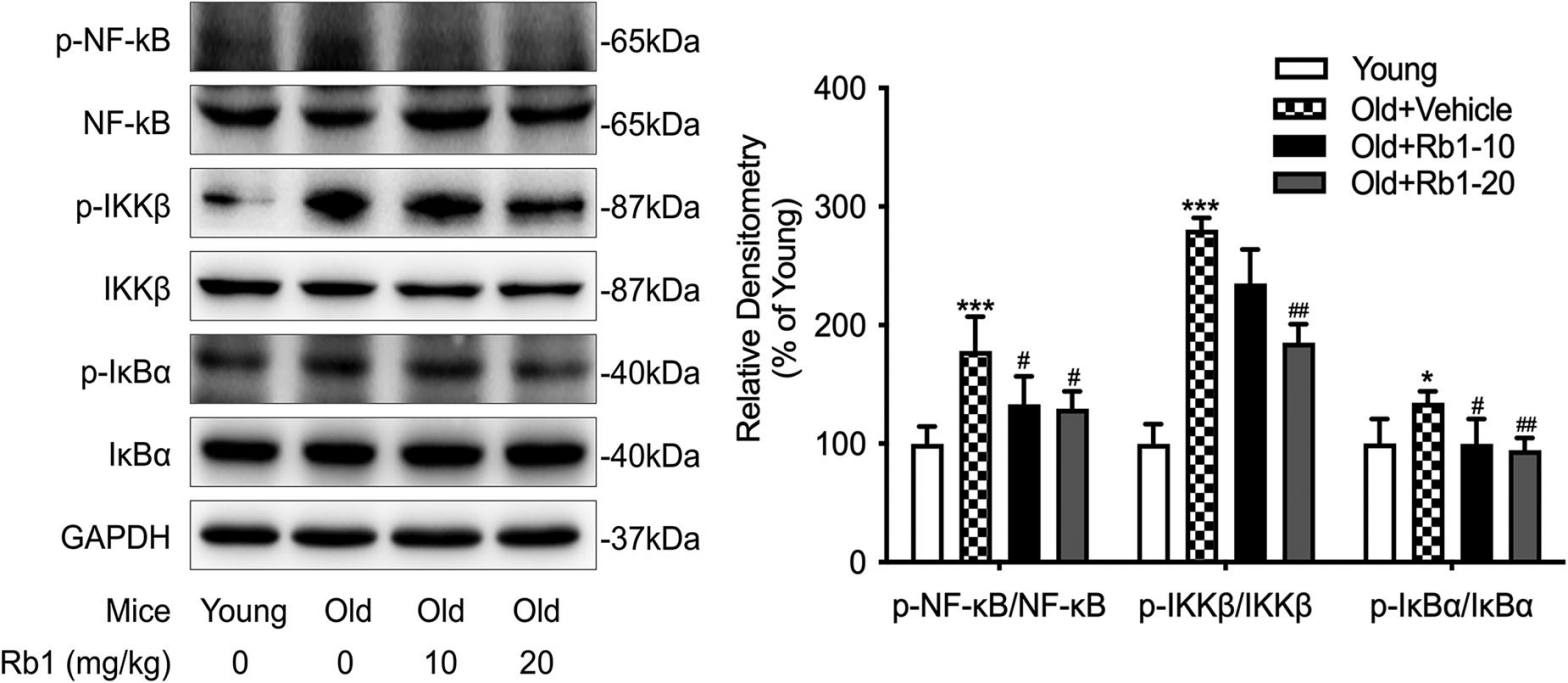
**Rb1 inhibits NF‐κB pathway activation**. Assessment of the phosphorylation levels of NF‐κB, IKKβ, and IKBα in the hippocampus of mice using Western blot analysis. The ratios of p‐NF‐κB/NF‐κB, p‐IKKβ/IKKβ, and p‐IκBα/IκBα were quantified for each group. *n* = 5 mice per group. Data are expressed as mean ± SD (^**^ *p* < 0.01 vs. the Young group; ^#^ *p* < 0.05, ^##^ *p* < 0.01 vs. the Old + Vehicle group). Statistical analysis was performed using one‐way ANOVA, followed by Tukey's multiple comparisons test.

The NF‐κB signaling pathway is critical to the inflammatory cascade response to various stimuli (Ridder and Schwaninger 2009). We therefore examined whether Rb1 ameliorates aging‐associated cognitive impairment and neuroinflammation by modulating NF‐κB signaling. The Western blot analysis showed that, compared with the young mice, the protein phosphorylation within the NF‐κB signaling pathways was prominently enhanced in the aging mice. Conversely, Rb1 administration substantially suppressed phosphorylation of NF‐κB, IKKβ, and IκBα in the aging mice (Figure 6), indicating that Rb1 potentially restrains NF‐κB pathway activation in aging mice.

## 4 Discussion

This study employed aged mice to examine the effects of Rb1 on aging‐induced cognitive dysfunction. We demonstrate that Rb1 alleviates aging‐related cognitive deficits by suppressing hippocampal neuroinflammation, a process linked to modulation of the NF‐κB signaling pathway. These findings suggest that Rb1 may be a potential therapeutic strategy for preventing aging‐related neurodegenerative disease.

Aging is an irreversible process and a fundamental risk factor for numerous neurodegenerative conditions, characterized by progressive declines in memory, attention, orientation, and broader cognitive abilities (Hou et al. 2019). Age‐related cognitive impairment constitutes the primary symptom of various neurodegenerative diseases affecting the elderly population (Shwe et al. 2018). Reports indicate that approximately 40% of individuals aged 65 and older experience some degree of cognitive decline without any overt pathology (Andrade and Radhakrishnan 2009). We used female mice for this study because they exhibit greater susceptibility to cognitive decline during normal aging, as well as an earlier onset and faster progression of neurodegenerative disease (Gao et al. 2016; Hebda‐Bauer et al. 2007). Our findings on aging‐related cognitive dysfunction in female C57BL/6 mice are consistent with existing evidence. Our results align with previous researches identifying Rb1 as a promising candidate for preventing cognitive deficits (Chen et al. 2019; Lin et al. 2019; Wang et al. 2018; Yang et al. 2020), demonstrating that Rb1 administration significantly improved spatial learning and memory performance in aged female mice, as assessed using the MWM test.

While essential for cognition, the hippocampus is highly vulnerable to age‐related decline. With advancing age, both the structural and functional integrity of the hippocampus are impaired (Geinisman et al. 1995). The accumulation of neurocellular damage within this structure represents a key pathological marker of aging that underlies cognitive impairment. Consequently, reducing neuronal damage is widely regarded as an effective therapeutic strategy for mitigating age‐related cognitive decline (Seman et al. 2023). A previous study showed that Rb1 pretreatment reverses hippocampal impairment in rats subjected to chronic restraint stress (Jiang et al. 2021). However, whether Rb1 protects against age‐related cognitive impairment—and its underlying molecular mechanisms—remains unclear. The present study found that Rb1 treatment reduces the expression of senescence‐related proteins and mitigates neuronal cell loss, indicating that Rb1 efficiently suppresses excessive senescence in the aging mouse hippocampus and could serve as a potential therapeutic agent for age‐related cognitive impairment.

Aging is associated with increased neuroinflammation, which critically contributes to the pathogenesis of age‐related neurodegenerative disease through the release of proinflammatory cytokines (López‐Otín et al. 2013). The hippocampus of aged animals exhibits microglial activation, characterized by a marked increase in several proinflammatory cytokines (Sadagurski et al. 2017). An excessive inflammatory response can initiate cerebral inflammation, induce neurotoxic effects, impair neurological function, ultimately leading to cognitive deficits (Kery et al. 2020). The anti‐inflammatory properties of Rb1 may represent a potential mechanism for alleviating aging‐induced cognitive impairments. Our findings are consistent with those of a previous study demonstrating aging‐induced hippocampal neuroinflammation in mice, as indicated by elevated levels of inflammatory cytokines, including IL‐6 and TNF‐α (Li et al. 2023). Notably, Rb1 administration reversed these alterations and reduced neuroinflammation in the hippocampus of aged mice, indicating that Rb1 mitigates hippocampal neuroinflammation and thereby creates a more favorable microenvironment for cognitive function. Future work should identify these cytokines’ specific cellular sources, such as microglia, astrocytes, or neurons, using techniques including double‐label immunofluorescence with cell‐specific markers and complementary cell‐based assays.

The question remains: How does Rb1 inhibit inflammation? Neuroinflammatory research has established that the NF‐κB signaling pathway contributes to neurodegenerative disease (Yuan et al. 2017). This classic inflammatory pathway drives the progression of various inflammatory conditions by controlling the expression of multiple target genes and regulating the transcription of proinflammatory mediators. Upon IKK activation, IκB undergoes phosphorylation and degradation, which releases NF‐κB. The phosphorylated, liberated NF‐κB translocates to the nucleus and rapidly induces transcription of its target genes (Dolatshahi et al. 2021). Activation of the NF‐κB pathway induces neuroinflammatory responses and triggers multiple nervous system diseases (Singh et al. 2020). Conversely, inhibiting NF‐kB suppresses the expression of proinflammatory genes, thereby reducing the overall inflammatory response in the brain and ultimately alleviating age‐related cognitive decline (Li et al. 2023; Liang et al. 2025). A previous study identified a compound naturally occurring in a medicinal plant that alleviates neurodegenerative disease by inhibiting NF‐κB‐mediated neuroinflammation (Li et al. 2025). Our earlier work demonstrated that Rb1 ameliorates aging‐related myocardial dysfunction potentially by suppressing the NF‐κB signaling pathway (Ke et al. 2020), suggesting that Rb1 may also alleviate age‐related neuroinflammation through modulation of this pathway. While direct evidence of NF‐κB nuclear translocation or DNA‐binding activity would provide a classical demonstration, the phosphorylation of IKKβ, IκBα, and NF‐κB itself represents crucial upstream steps in its activation (Dolatshahi et al. 2021). Consequently, we assessed NF‐κB activation based on these established phosphorylation markers in hippocampal lysates. Our results showed that NF‐κB, IKKβ, and IκBα phosphorylation levels were markedly elevated in the hippocampus of aged mice, indicating NF‐κB pathway activation, consistent with prior research (Cheng et al. 2021). Rb1 administration reversed the phosphorylation of these proteins in aging mice, suggesting that Rb1 may repress the NF‐κB pathway during aging.

## 5 Conclusion

This study demonstrated, for the first time, that Rb1 ameliorates hippocampal structural damage and attenuates the inflammatory response, thereby alleviating aging‐related cognitive impairment, at least in part by inhibiting the NF‐κB signaling pathway. Given the inevitability of aging, it is critical to identify agents that can prevent or treat age‐related impairment of hippocampal cells. Our findings validate the beneficial effects of Rb1 on cognitive deficits in aging mice. One limitation of this study is that we examined the effects of Rb1 only in a female mouse model of aging‐related cognitive decline. Further investigations using in vitro models, NF‐κB signaling inhibition experiments, and other mammalian species are therefore warranted. Furthermore, sex differences in neuroinflammatory responses and cognitive outcomes may exist; therefore, future studies should include both male and female animals to determine the full translational relevance of our findings.

## Author Contributions

**Shi‐Ye Ke**: conceiving and designing the study and critically revising the manuscript. **Zhi‐Gang Chen**: collecting the data. **Wei‐Feng Lu**: analyzing and interpreting the data and critically revising the manuscript. **Yu‐Zhen Zhang**: analyzing and interpreting the data. **Qun‐Lin Xiao**: drafting the manuscript.

## Funding

This research was funded by the National Natural Science Foundation of China (No. 82304951), and the Natural Science Foundation of Shenzhen, China (No. JCYJ20240813150604007).

## Ethics Statement

This study was approved by the Institutional Animal Care and Use Committee (IACUC) of Sun Yat‐Sen University (Ethics Approval Code: SYSU‐IACUC‐2019‐000139).

## Conflicts of Interest

The authors declare no conflicts of interest.

## Data Availability

The data that support the findings of this study are available on request from the corresponding author.

## References

[brb371334-bib-0001] Andrade, C. , and R. Radhakrishnan . 2009. “The Prevention and Treatment of Cognitive Decline and Dementia: An Overview of Recent Research on Experimental Treatments.” Indian Journal of Psychiatry 51: 12. 10.4103/0019-5545.44900. 19742190 PMC2738400

[brb371334-bib-0002] Bishop, N. A. , T. Lu , and B. A. Yankner . 2010. “Neural Mechanisms of Ageing and Cognitive Decline.” Nature 464: 529–535. 10.1038/nature08983. 20336135 PMC2927852

[brb371334-bib-0003] Chen, C. , H. Zhang , H. Xu , Y. Zheng , T. Wu , and Y. Lian . 2019. “Ginsenoside Rb1 Ameliorates Cisplatin‐Induced Learning and Memory Impairments.” Journal of Ginseng Research 43: 499–507. 10.1016/j.jgr.2017.07.009. 31695559 PMC6823748

[brb371334-bib-0004] Cheng, J. , R. Zhang , Z. Xu , et al. 2021. “Early Glycolytic Reprogramming Controls Microglial Inflammatory Activation.” Journal of Neuroinflammation 18: 129. 10.1186/s12974-021-02187-y. 34107997 PMC8191212

[brb371334-bib-0005] Dahan, L. , C. Rampon , and C. Florian . 2020. “Age‐Related Memory Decline, Dysfunction of the Hippocampus and Therapeutic Opportunities.” Progress in Neuro‐Psychopharmacology & Biological Psychiatry 102: 109943. 10.1016/j.pnpbp.2020.109943. 32298784

[brb371334-bib-0006] Dolatshahi, M. , M. H. Ranjbar Hameghavandi , M. Sabahi , and S. Rostamkhani . 2021. “Nuclear Factor‐Kappa B (NF‐κB) in Pathophysiology of Parkinson Disease: Diverse Patterns and Mechanisms Contributing to Neurodegeneration.” European Journal of Neuroscience 54: 4101–4123. 10.1111/ejn.15242. 33884689

[brb371334-bib-0007] Gao, H. , P. Yan , S. Zhang , et al. 2016. “Chronic Alpha‐Linolenic Acid Treatment Alleviates Age‐Associated Neuropathology: Roles of PERK/eIF2alpha Signaling Pathway.” Brain, Behavior, and Immunity 57: 314–325. 10.1016/j.bbi.2015.09.012. 26399745

[brb371334-bib-0008] Geinisman, Y. , L. Detoledo‐Morrell , F. Morrell , and R. E. Heller . 1995. “Hippocampal Markers of Age‐Related Memory Dysfunction: Behavioral, Electrophysiological and Morphological Perspectives.” Progress in Neurobiology 45: 223–252. 10.1016/0301-0082(94)00047-L. 7777673

[brb371334-bib-0009] Haider, S. , and S. Tabassum . 2018. “Impact of 1‐Day and 4‐Day MWM Training Techniques on Oxidative and Neurochemical Profile in Rat Brain: A Comparative Study on Learning and Memory Functions.” Neurobiology of Learning and Memory 155: 390–402. 10.1016/j.nlm.2018.09.003. 30195048

[brb371334-bib-0010] Hebda‐Bauer, E. K. , J. Luo , S. J. Watson , and H. Akil . 2007. “Female CREBalphadelta‐Deficient Mice Show Earlier Age‐Related Cognitive Deficits Than Males.” Neuroscience 150: 260–272. 10.1016/j.neuroscience.2007.09.019. 18029102 PMC2278026

[brb371334-bib-0011] Hou, Y. , X. Dan , M. Babbar , et al. 2019. “Ageing as a Risk Factor for Neurodegenerative Disease.” Nature Reviews Neurology 15: 565–581. 10.1038/s41582-019-0244-7. 31501588

[brb371334-bib-0012] Hsu, J. J. , J. Lu , S. Umar , et al. 2018. “Effects of Teriparatide on Morphology of Aortic Calcification in Aged Hyperlipidemic Mice.” American Journal of Physiology. Heart and Circulatory Physiology 314: H1203–H1213. 10.1152/ajpheart.00718.2017. 29451816 PMC6032086

[brb371334-bib-0013] Huang, R. , J. Li , J. Xiao , et al. 2025. “Prenatal Stress Increases Learning and Memory Deficits in Offspring: A Toxicological Study on Hippocampal Neuronal Damage in Rats.” Ecotoxicology and Environmental Safety 295: 118167. 10.1016/j.ecoenv.2025.118167. 40215686

[brb371334-bib-0014] Jiang, N. , K. Wang , Y. Zhang , et al. 2021. “Protective Effect of Ginsenoside Rb1 Against Chronic Restraint Stress (CRS)‐Induced Memory Impairments in Rats.” Behavioural Brain Research 405: 113146. 10.1016/j.bbr.2021.113146. 33545198

[brb371334-bib-0015] Ke, S. Y. , D. H. Liu , L. Wu , et al. 2020. “Ginsenoside Rb1 Ameliorates Age‐Related Myocardial Dysfunction by Regulating the NF‐κB Signaling Pathway.” American Journal of Chinese Medicine 48: 1369–1383. 10.1142/S0192415X20500676. 32933311

[brb371334-bib-0016] Kempster, P. , and A. Ma . 2022. “Parkinson's Disease, Dopaminergic Drugs and the Plant World.” Frontiers in Pharmacology 13: 970714. 10.3389/fphar.2022.970714. 36133818 PMC9483127

[brb371334-bib-0017] Kery, R. , A. P. F. Chen , and G. W. Kirschen . 2020. “Genetic Targeting of Astrocytes to Combat Neurodegenerative Disease.” Neural Regeneration Research 15: 199–211. 31552885 10.4103/1673-5374.265541 PMC6905329

[brb371334-bib-0018] Kwon, S. , M. Iba , C. Kim , and E. Masliah . 2020. “Immunotherapies for Aging‐Related Neurodegenerative Diseases‐Emerging Perspectives and New Targets.” Neurotherapeutics 17: 935–954. 10.1007/s13311-020-00853-2. 32347461 PMC7222955

[brb371334-bib-0019] Kwon, H. S. , and S. H. Koh . 2020. “Neuroinflammation in Neurodegenerative Disorders: The Roles of Microglia and Astrocytes.” Translational Neurodegeneration 9: 42. 10.1186/s40035-020-00221-2. 33239064 PMC7689983

[brb371334-bib-0020] Lawrence, T. 2009. “The Nuclear Factor NF‐kappaB Pathway in Inflammation.” Cold Spring Harbor Perspectives in Biology 1: a001651. 10.1101/cshperspect.a001651. 20457564 PMC2882124

[brb371334-bib-0021] Li, X. , Y. Gao , X. Han , et al. 2023. “Maresin1 Ameliorates Postoperative Cognitive Dysfunction in Aged Rats by Potentially Regulating the NF‐κB Pathway to Inhibit Astrocyte Activation.” Experimental Gerontology 176: 112168. 10.1016/j.exger.2023.112168. 37055002

[brb371334-bib-0022] Li, X. L. , Z. H. Lin , S. R. Chen , et al. 2025. “Tiaogeng Decoction Improves Mild Cognitive Impairment in Menopausal APP/PS1 Mice Through the ERs/NF‐κ b/AQP1 Signaling Pathway.” Phytomedicine 138: 156391. 10.1016/j.phymed.2025.156391. 39848022

[brb371334-bib-0023] Liang, A. , L. Zhang , J. Peng , et al. 2025. “Human Umbilical Cord Mesenchymal Stem Cells Ameliorate Cognitive Decline by Restoring Senescent Microglial Function via NF‐kappaB‐SREBP1 Pathway Inhibition.” Aging Cell 24: e70259. 10.1111/acel.70259. 41078306 PMC12686588

[brb371334-bib-0024] Lin, J. , S. Gao , T. Wang , et al. 2019. “Ginsenoside Rb1 Improves Learning and Memory Ability Through Its Anti‐inflammatory Effect in Aβ(1‐40) Induced Alzheimer's Disease of Rats.” American Journal of Translational Research 11: 2955–2968. 31217866 PMC6556649

[brb371334-bib-0025] López‐Otín, C. , M. A. Blasco , L. Partridge , M. Serrano , and G. Kroemer . 2013. “The Hallmarks of Aging.” Cell 153: 1194–1217. 23746838 10.1016/j.cell.2013.05.039 PMC3836174

[brb371334-bib-0026] Martínez de Toda, I. , N. Ceprián , E. Díaz‐Del Cerro , and M. De la Fuente . 2021. “The Role of Immune Cells in Oxi‐Inflamm‐Aging.” Cells 10: 2974. 34831197 10.3390/cells10112974 PMC8616159

[brb371334-bib-0027] Qian, H. , F. Gao , X. Wu , et al. 2023. “Activation of the CD200/CD200R1 Axis Attenuates Neuroinflammation and Improves Postoperative Cognitive Dysfunction via the PI3K/Akt/NF‐kappaB Signaling Pathway in Aged Mice.” Inflammation Research 72: 2127–2144. 10.1007/s00011-023-01804-1. 37902837

[brb371334-bib-0028] Qin, Q. , H. Mehta , K. Yen , et al. 2018. “Chronic Treatment With the Mitochondrial Peptide Humanin Prevents Age‐Related Myocardial Fibrosis in Mice.” American Journal of Physiology. Heart and Circulatory Physiology 315: H1127–H1136. 10.1152/ajpheart.00685.2017. 30004252 PMC6415743

[brb371334-bib-0029] Rauf, A. , and M. M. Rahman . 2022. “Potential Therapeutics Against Neurological Disorders: Natural Products‐Based Drugs.” Frontiers in Pharmacology 13: 950457. 10.3389/fphar.2022.950457. 36060010 PMC9437920

[brb371334-bib-0030] Ridder, D. A. , and M. Schwaninger . 2009. “NF‐kappaB Signaling in Cerebral Ischemia.” Neuroscience 158: 995–1006. 10.1016/j.neuroscience.2008.07.007. 18675321

[brb371334-bib-0031] Sadagurski, M. , G. Cady , and R. A. Miller . 2017. “Anti‐Aging Drugs Reduce Hypothalamic Inflammation in a Sex‐Specific Manner.” Aging Cell 16: 652–660. 10.1111/acel.12590. 28544365 PMC5506421

[brb371334-bib-0032] Seman, A. , P. K. Chandra , S. D. Byrum , et al. 2023. “Targeting Mitochondria in the Aged Cerebral Vasculature With SS‐31, a Proteomic Study of Brain Microvessels.” Geroscience 45: 2951–2965. 10.1007/s11357-023-00845-y. 37458933 PMC10643806

[brb371334-bib-0033] Shwe, T. , W. Pratchayasakul , N. Chattipakorn , and S. C. Chattipakorn . 2018. “Role of D‐Galactose‐Induced Brain Aging and Its Potential Used for Therapeutic Interventions.” Experimental Gerontology 101: 13–36. 10.1016/j.exger.2017.10.029. 29129736

[brb371334-bib-0034] Singh, S. S. , S. N. Rai , H. Birla , W. Zahra , A. S. Rathore , and S. P. Singh . 2020. “NF‐κB‐Mediated Neuroinflammation in Parkinson's Disease and Potential Therapeutic Effect of Polyphenols.” Neurotoxicity Research 37: 491–507. 10.1007/s12640-019-00147-2. 31823227

[brb371334-bib-0035] Wang, Y. , Y. Li , W. Yang , et al. 2018. “Ginsenoside Rb1 Inhibit Apoptosis in Rat Model of Alzheimer's Disease Induced by Abeta(1‐40).” American Journal of Translational Research 10: 796–805. 29636869 PMC5883120

[brb371334-bib-0036] Yang, Y. , S. Li , H. Huang , et al. 2020. “Comparison of the Protective Effects of Ginsenosides Rb1 and Rg1 on Improving Cognitive Deficits in SAMP8 Mice Based on Anti‐Neuroinflammation Mechanism.” Frontiers in Pharmacology 11: 834. 10.3389/fphar.2020.00834. 32587516 PMC7298198

[brb371334-bib-0037] Yang, D. , C. Xiao , F. Long , et al. 2019. “Fra‐1 Plays a Critical Role in Angiotensin II‐Induced Vascular Senescence.” Faseb Journal 33: 7603–7614. 10.1096/fj.201801671RRRR. 30892941

[brb371334-bib-0038] Yu, C. , Y. Zhang , Y. Guo , et al. 2024. “Ginsenoside Rb1 Attenuates Mouse Cerebral Ischemia/Reperfusion Induced Neurological Impairments Through Modulation of Microglial Polarization.” Folia Neuropathologica 62: 215–222. 10.5114/fn.2024.134300. 39165207

[brb371334-bib-0039] Yuan, J. , W. Liu , H. Zhu , et al. 2017. “Curcumin Inhibits Glial Scar Formation by Suppressing Astrocyte‐Induced Inflammation and Fibrosis in Vitro and in Vivo.” Brain Research 1655: 90–103. 10.1016/j.brainres.2016.11.002. 27865778

[brb371334-bib-0040] Zheng, W. , W. Wu , Y. Li , et al. 2025. “Lycorine Pre‐Treatment Alleviates Microglia Inflammation After Cerebral Ischemia by Inhibiting NF‐κB Phosphorylation.” Brain Sciences 15: 290. 10.3390/brainsci15030290. 40149811 PMC11939849

[brb371334-bib-0041] Zhou, P. , W. Xie , Y. Sun , et al. 2019. “Ginsenoside Rb1 and Mitochondria: A Short Review of the Literature.” Molecular and Cellular Probes 43: 1–5. 10.1016/j.mcp.2018.12.001. 30529056

